# Unmet need for alcohol use disorder treatment in reproductive-age females, with emphasis on pregnant and parenting populations in the United States: Findings from NSDUH 2015–2021

**DOI:** 10.1371/journal.pone.0301810

**Published:** 2024-04-09

**Authors:** Anna Shchetinina, Natalie Slopen

**Affiliations:** Department of Social and Behavioral Sciences, Harvard T.H. Chan School of Public Health, Boston, Massachusetts, United States of America; University of Connecticut Health Center: UConn Health, UNITED STATES

## Abstract

The negative effects of alcohol use can transmit intergenerational harm if alcohol use disorder (AUD) occurs during pregnancy and/or while parenting a child. Prenatal alcohol exposure is the leading preventable cause of congenital anomalies in the USA, and heavy drinking in women has been on the rise, further accelerated by the COVID-19 pandemic. This study describes the most recent patterns in the past year AUD prevalence and treatment among reproductive-aged women, with a specific focus on pregnant and parenting women, and barriers to treatment among those affected. We analyzed data on reproductive-age women from the National Survey on Drug Use and Health (2015–2021). We used generalized linear models to estimate prevalence ratios (PR) for past 12-month AUD and its treatment based on DSM-V criteria. We considered sociodemographic characteristics, including age, race/ethnicity, income, health insurance type, and arrest history. Pregnant and parenting women displayed lower risk for AUD (PR = 0.48, 95% CI:0.41–0.57; PR = 0.5 95% CI:0.48–0.54, respectively) relative to non-pregnant/non-parenting women. Excess risk for AUD was associated with education (some college vs. college graduates, PR = 1.07, 95% CI:1.01–1.13) and history of arrests (PR = 2.93, 95% CI:2.67–3.21). There were no clear differences in AUD treatment use based on parenting or pregnancy status. Among those with AUD, the prevalence of treatment was higher among individuals aged 35–49 years compared to those 18–25 years (PR = 1.6, 95% CI: 1.19–2.14) and in those enrolled in Medicaid vs. private insurance (PR = 2.62, 95%CI:1.97–3.47). Financial barriers and treatment not being a priority were the most frequently reported barriers to treatment. To promote well-being among parents and their children, healthcare providers should prioritize reproductive-age women at higher AUD risk. Decreasing the stigma attached to AUD and intensifying efforts to educate women about the dangers of AUD may improve treatment use among pregnant and parenting women.

## Introduction

An estimated one-third of people suffering from alcohol use disorder (AUD) worldwide are women [1], with AUD characterized by psychological or physical dependence on alcohol. Generally, women are more susceptible to the immediate and long-term negative effects of AUD, including but not limited to liver and cardiovascular disease, breast and other cancers, sexual violence, and mental health problems [2]. The negative effects of alcohol misuse can transmit intergenerational harm if AUD occurs during pregnancy and/or while parenting a child. Prenatal alcohol exposure is the leading preventable cause of congenital anomalies in the United States [3]. Fetal alcohol exposure can cause various health consequences, such as fetal alcohol spectrum disorder, withdrawal syndrome, low birth weight, neurodevelopmental problems, as well as preterm birth, stillbirth, and miscarriage [1]. AUD is not typically limited to pregnancy, and parental AUD is a known risk factor for compromised parenting [4], adverse childhood experiences, and a host of health problems across development, including excess weight gain, poor academic skills, aggressiveness, and substance misuse [5]. The prevalence of binge and heavy drinking among women with and without children increased between 2006 and 2018 [6], despite the growing body of research on the harms associated with alcohol consumption during pregnancy and parenting. Furthermore, according to Boschuetz et al. [7], the COVID-19 pandemic led to a notable rise in high-risk alcohol consumption among women. This study found that during social distancing, females were more likely to develop high-risk behaviors than males (63% of female respondents developed high-risk behavior vs 25% of male respondents). The frequency and quantity of alcohol intake in women also increased significantly. Additionally, women who were parents or had a previous history of substance abuse faced an elevated risk of engaging in alcohol use [7].

The present study provides updated data on the past 12-month prevalence of AUD and treatment for AUD among women in the United States, and barriers to treatment among those affected. Historically, women who consume alcohol have been subject to more stigmatization than men, which likely undermines recovery efforts [8]. A prospective study including 2,592 adults meeting criteria for alcohol abuse followed for approximately 3 years (2000–2001 to 2004–2005) found that women were half as likely to use alcohol dependence treatment services relative to men, and were also more likely than men to think that improvement in their drinking would happen without help, and therefore were less likely to seek treatment [9]. A study using national survey data from 2007 to 2014 revealed significant disparities in access to treatment for pregnant and parenting women, with lower rates of treatment among Black and Hispanic women relative to white women (adjusted OR = 0.3, 95% CI: 0.2–0.5 and OR = 0.6; 95% CI 0.4–0.9, respectively) [10]. Importantly, few studies have documented the prevalence of AUD and unmet treatment needs using updated clinical criteria for AUD according to the Diagnostic and Statistical Manual of Mental Disorders 5 (DSM-5) or used recent national data to examine barriers to AUD treatment among pregnant and parenting individuals in the U.S.

The current project builds on earlier findings using data from the National Survey of Drug Use and Health (NSDUH), a large, nationally representative sample, and uses established DSM-5 diagnostic criteria to define AUD to examine the association between AUD over the previous 12 months and received treatment. We focus our attention on the period from 2015 to 2021 for the following reasons. First, we aim to summarize the most recent data, building upon a previously published study that identified unmet treatment requirements among pregnant and non-pregnant women from the years 2007 to 2014, as documented by Martin et al. in 2020 [10]. Second, the NSDUH substance use survey methodology made substantial changes in 2015, thus impeding comparisons of estimates before and after 2015. On this basis, we draw on 2015 to 2021 data to update the prevalence of past 12-month AUD among reproductive-aged women in the US, providing estimates for three distinct groups of women: pregnant, parenting, and not pregnant not parenting. We also evaluate unmet AUD treatment needs in this population and assess the associated demographic characteristics. Finally, we describe barriers to care and their variability across the groups.

## Methods

The data for this study were obtained from the NSDUH surveys conducted between 2015 and 2021. These surveys, sponsored by the Substance Abuse and Mental Health Services Administration, gather nationally representative data on substance use in the United States utilizing a multistage probability sampling to select households or non-institutional group homes from the 50 states and the District of Columbia, followed by the selection of individuals aged 12 years and older. Sampling weights account for non-response and oversampling of specific demographic groups, such as young adults and Black and Hispanic respondents to align with population estimates from the U.S. Census Bureau. The response rates for the surveys ranged from 53 to 75 percent during the years under study [11]. The surveys were conducted by trained interviewers using computer-assisted personal interviewing and audio computer-assisted self-interviewing methods. The interview methods were designed to handle sensitive subjects, maintain privacy and confidentiality, and encourage participants to provide truthful responses, especially regarding substance use [11]. Prior to the interviews, informed consent was obtained from all individuals involved. As our study used secondary data, there was no primary recruitment of participants and obtaining consent from individuals included in this study was not feasible. Our study is exempt from Institutional Review Board approval as it solely utilized publicly available deidentified data.

The sample for this study was limited to reproductive-age women, defined as aged 18–49 years. We categorized eligible respondents as “Pregnant”, “Not pregnant, Parenting” and “Not Pregnant, Not Parenting”. Pregnant women were identified based on their response of “yes” to the question, “Are you currently pregnant.” The “Not Pregnant, Parenting” category was identified if the reported number of children in the household was one or more and they did not report a current pregnancy. Those women who were not included in the two groups but were in the relevant age range were categorized as “Not Pregnant, Not Parenting”. Women within the age range of 18–49 who had missing information regarding the presence or absence of AUD in the past year were excluded from the analysis (n = 1022), resulting in a final sample of 120,110 individuals. The proportion of women aged 18–49 with missing AUD status was less than 1 percent. Missing values for most variables were imputed using standard imputation procedures by NSDUH in the post-interview data handling [11].

### Measures

#### AUD

A variable for the past year AUD was constructed based on the criteria outlined in the DSM-5. As NSDUH only began implementing DSM-5 in 2020, we manually constructed a variable for the years 2015–2019 using responses to questions that represented the DSM-5 criteria. We followed the approach described by Parsley et al. [12] to create this variable (details can be found in S1 Table). Consistent with other research, to construct a dichotomous AUD outcome, we coded individuals who met two or more criteria as positive for AUD, while those who met less than 2 criteria were coded as negative for AUD. This dichotomization aligned with the diagnostic criteria specified in the DSM-5. Starting in 2020, NSDUH incorporated a DSM-5-consistent AUD variable.

#### AUD treatment

In this analysis, AUD treatment was determined based on a question of whether the respondents had received treatment or counseling for their alcohol or drug use, excluding cigarettes, in the past 12 months. The alcohol treatment variable was further specified with the response to the question “Was the treatment for alcohol use only, drug use only, or both alcohol and drug use?”. Treatment could have been received at various locations, including hospitals, rehabilitation facilities, mental health centers, private physician’s offices, substance abuse-related emergency rooms, or self-help groups.

#### Barriers to treatment

The NSDUH asked questions about barriers to treatment in the women with a past year AUD treatment need that was not met. Respondents were asked the question, "Which of these statements explains why you did not seek the treatment/additional treatment you needed for your use of alcohol?". They were provided with 14 options and could select one or more reasons for not receiving treatment. Following the approach taken by Ali et al. [13], we categorized the responses into four groups: financial barriers, access barriers, stigma, and treatment not being a priority (details can be found in S2 Table). “Financial barriers” refer to individuals who did not receive treatment because they could not afford the cost or because their insurance did not cover it. “Access barriers” encompassed reasons such as lack of transportation, unavailability of the specific treatment needed, no openings in programs, or not knowing where to seek treatment. “Stigma” refers to individuals who choose not to receive treatment due to concerns about negative opinions from neighbors, not wanting others to know, or fearing negative effects on their job. Last, “Treatment not a priority” includes those who believed they could handle the problem without treatment, did not have time for it, did not think treatment would help or were not prepared to stop using alcohol. Due to constraints in sample size, we grouped pregnant and parenting women into a single category for the analysis of barriers to treatment, distinguishing between two groups: parenting/pregnant and not parenting, not pregnant women.

#### Sociodemographic variables

The study incorporated several sociodemographic risk factors, including age categories (18–25, 26–34, 35–49), racial/ethnic background (non-Hispanic White, non-Hispanic Black, Hispanic, Other), educational attainment (less than high school, high school, some college or an associate degree, college graduate), family income ($0–19,999, $20,000–49,999, $50,000–74,999, ≥$75,000), geographical area type (large metro, small metro, non-metro), health insurance type (private, Medicaid/CHIP, Medicare, other or no insurance), arrest history, and a history of major depressive episode in the past year. In addition, all models included a variable with survey year.

### Statistical analysis

All statistical analyses were performed in RStudio 2022.12.0+353, taking into account the complex survey design by applying sample weights. Consistent with previous studies [14, 15], the sample weights were divided by the number of concatenated datasets. The prevalence of AUD and treatment outcomes, as well as sociodemographic risk factors, were calculated for the three groups of interest. Generalized linear models (glm) with Poisson and log link were utilized to assess the association between sociodemographic factors and AUD among the three categories of women. We first ran the bivariate models, then proceeded with adjustment for non-socioeconomic status variables, and finished with fully adjusted models that included non-socioeconomic and socioeconomic variables. The models were additionally adjusted for the year of response recording. Similarly, glm with Poisson and log link was employed to examine the association with AUD treatment among those categorized as positive for the disorder. The results were reported as adjusted prevalence ratios (aPR). Due to the impact of the COVID-19 pandemic on the NSDUH data collection in 2020–2021, we have conducted an additional sensitivity analysis that included the data from 2015 to 2019 only (S3 and S4 Tables). There were no missing data in the demographic variables, and for the outcome variables, a complete case analysis was conducted.

## Results

### Sample characteristics

Our analytic sample consisted of 4,573 (3.2% weighted) pregnant women, 53,045 parenting women (49.7%), and 62,492 women who were not pregnant and not parenting (47.1%) (see Table 1; total n = 120,110, weighted N = 66,519,300). The majority in each category had more than a high school education, lived in the metro area, and had private health insurance. Among the pregnant category, 52.8% were in the 26–34 years age group, and 30.1% of the sample were 18–25 years. The age distribution was older in the parenting group, where the majority (59.9%) were 35–49 or 26–34 (31.7%). Approximately 6.3% of pregnant and 6.6% of parenting women reported having AUD in the past year, whereas the prevalence in not pregnant, not parenting women was 13%.

**Table 1:** Demographic characteristics of the study sample (with the survey-weighted percentage in the brackets).

	Not pregnant, not parenting (n = 62492, weighted N = 31,326,025, 47.1%)	Pregnant (n = 4,573, weighted N = 2,113,723, 3.2%)	Not pregnant, parenting (n = 53045, weighted N = 33,079,552, 49.7%)	p-value
**Age (years)**				<0.001
18–25	35,816 (42.2%)	2,096 (30.1%)	9223 (8.4%)	
26–34	12,140 (25%)	1,958 (52.8%)	17,669 (31.7%)	
35–49	14,536 (32.8%)	519 (17.1%)	26,153 (59.9%)	
**Education**				<0.001
Less than High School	5,748 (8.8%)	621 (12.1%)	6299 (11.4%)	
High School	13,800 (21.2%)	1,217 (22.8%)	11948 (20.4%)	
Some College/Associate Degree	23,599 (36.2%)	1388 (29.5%)	18101 (33.6%)	
College Graduate	19,345 (33.8%)	1347 (35.6%)	16697 (34.6%)	
**Race/Ethnicity**				<0.001
White	36,580 (58.3%)	2506 (54.8%)	29864 (54.4%)	
Black/African American	7,858 (13.6%)	682 (15.6%)	7017 (13.7%)	
Hispanic	10,618 (17.4%)	892 (19.6%)	11005 (22.4%)	
Other	7,436 (10.6%)	493 (10.1%)	5159 (9.5%)	
**Residence area type**				<0.001
Large Metro	29,769 (59.4%)	1900 (55.4%)	22955 (56.2%)	
Small Metro	22,403 (29.2%)	1699 (30.4%)	19427 (30.1%)	
Non-Metro	10,320 (11.4%)	974 (14.3%)	10663 (13.7%)	
**Annual Household Income**				<0.001
Less than $20,000	15,246 (21%)	1033 (19.1%)	5533866.8 (16.7%)	
$20,000-$49,999	18,903 (29.4%)	1535 (30.6%)	9445934.9 (28.6%)	
$50,000-$74,999	9,316 (15.5%)	659 (15.6%)	4922338.2 (14.9%)	
$75,000+	19,027 (34%)	1346 (34.7%)	13177411.9 (39.8%)	
**Health Insurance**				<0.001
Private	40,775 (65.4%)	2255 (53.5%)	30581 (60.8%)	
Medicaid/CHIP	9,763 (14.5%)	1666 (33.5%)	13338 (22.1%)	
Medicare	1,210 (2.3%)	44 (0.9%)	748 (1.4%)	
Other	3,474 (4.7%)	289 (5%)	5800 (4.2%)	
No insurance	7,270 (13.1%)	319 (7.1%)	5800 (11.5%)	
**Past year AUD (DSM_V)**				<0.001
Yes	8,421 (13%)	332 (6.3%)	3602 (6.6%)	
No	53,488 (87%)	4200 (93.7%)	49045 (93.5%)	
**Past year MDE**	10,753 (16.4%)	384 (7.6%)	5,202 (8.7%)	<0.001
**Year**				<0.001
2015–2016	18,321 (27.6%)	1469 (30%)	16879 (30.2%)	
2017–2018	17,800 (28.3%)	1441 (31.4%)	16459 (29.6%)	
2019–2020	15,572 (29.3%)	1005 (25.9%)	12247 (27.1%)	
2021	10,799 (14.8%)	658 (12.7%)	7460 (13.1%)	

AUD = Alcohol use disorder, DSM = Diagnostic and Statistical Manual of Mental Disorders, MDE = major depressive episode.

Among those who reported past-year AUD, pregnant and parenting women had a higher prevalence of treatment relative to the not pregnant, not parenting women (see Table 2). Treatment was modestly patterned by age, with the oldest group age (35–49) most likely to receive treatment (5.3%, 95%CI: 4.3–6.4), followed by the middle age group (ages 26–34 years; 4.9%, 95%CI: 3.8–6.1) and then the youngest (ages 18–25; 3.3.%, 95%CI: 2.6–4). Women with less than high school education and those in the lowest annual household income category had a higher treatment prevalence (8.0%, 95%CI: 5.8–10.2 and 7.1%, 95%CI: 5.4–8.8 respectively) relative to their counterparts with higher education or household incomes. The prevalence of past-year AUD in reproductive-age women increased from 8.5 percent (95%CI: 8.1–8.9) in 2015–2016 to 12.3 percent (95%CI: 11.9–13.5) in 2021, while the prevalence of treatment appears to decrease in 2019–2021 compared to earlier years (3.7%, 95%CI: 2.2–5.2 in 2021 and 4.8%, 95%CI: 3.8–5.8 in 2015–2016), although the estimates are not precise.

**Table 2:** Prevalence of treatment for AUD by need and demographics among women 18–49 years.

	AUD prevalence among females 18–49% (95%CI) (raw n = 12703) (weighted n = 6,489,162)	% Received treatment	
		Yes	No
		%(95%CI)	%(95%CI)
		(raw n = 576; 4.5%)	(raw n = 12127; 95.5%)
**Parenting status**			
Not Pregnant, Not Parenting	12.9% (12.5–13.4)	4.2% (3.6–4.8)	95.8% (95.2–96.5)
Pregnant	6.3% (5.3–7.3)	5.2% (1.6–8.9)	94.8% (91.1–98.5)
Not Pregnant, Parenting	6.6% (6.2–6.9)	5% (3.9–6.1)	95% (93.9–96.1)
**Age**			
18–25	13.9% (13.4–14.3)	3.3% (2.6–4)	96.7% (96–97.4)
26–34	10% (9.4–10.5)	4.9% (3.8–6.1)	95.1% (94–96.2)
35–49	7% (6.7–7.3)	5.3% (4.3–6.4)	94.7% (93.7–95.7)
**Race/Ethnicity**			
White	10.8% (10.4–11.2)	4.8% (4.1–5.5)	95.2% (94.5–96)
Black/African American	8.6% (8.1–9.2)	4.2% (2.8–5.5)	95.8% (94.5–97.2)
Hispanic	7.5% (7–8.1)	4.2% (2.9–5.5)	95.8% (94.5–97.1)
Other	8.1% (7.1–9)	3.1% (2.2–4)	96.9% (96–97.8)
**Residence area type**			
Large Metro	10% (9.6–10.4)	3.9% (3.4–4.5)	96.1% (95.5–96.6)
Small Metro	9.5% (9–9.9)	5.2% (4.1–6.3)	94.8% (93.7–95.9)
Non-Metro	7.7% (7.2–8.3)	5.6% (3.4–7.9)	94.4% (92.1–96.6)
**Education**			
Less than High School	6.3% (5.6–7)	8% (5.8–10.2)	92% (89.9–94.2)
High School	8.7% (8.2–9.2)	5.4% (4–6.8)	94.6% (93.2–96)
Some College/Associate Degree	10.6% (10.2–11.1)	5% (4.2–5.9)	95% (94.1–95.8)
College Graduate	10% (9.5–10.5)	2.8% (2–3.6)	97.2% (96.5–98)
**Annual Household Income**			
Less than $20,000	10.7% (10–11.3)	7.1% (5.4–8.8)	92.9% (91.2–94.6)
$20,000-$49,999	9.8% (9.4–10.2)	4.5% (3.4–5.6)	95.5% (94,4–96.7)
$50,000-$74,999	9.4% (8.7–10.3)	4.1% (2.9–5.3)	95.9% (94.7–97.1)
$75,000+	9% (8.6–9.3)	3.1% (2.5–3.7)	96.9% (96.3–97.5)
**Health insurance**			
Private	9.9% (9.6–10.1)	3.2% (2.7–3.7)	96.8% (96.3–97.4)
Medicaid/CHIP	8.7% (8.1–9.4)	8.3% (6.6–9.9)	91.7% (90.1–93.4)
Medicare	6.8% (4.8–8.7)	5% (1–9.4)	95% (90.6–99.3)
Other	10% (8.9–11.5)	4.3% (2.6–6.1)	95.7% (93.9–97.4)
No insurance	9.6% (8.9–10.3)	6.2% (4–8.4)	93.8% (91.6–96)
**Pasr-year MDE**	20.3% (19.3–21.3)	6.8% (5.5–8)	93.2% (92–94.5)
**Year**			
2015–2016	8.5% (8.1–8.9)	4.8% (3.8–5.8)	95.2% (94.2–96.2)
2017–2018	8.2% (7.8–8.6)	5.5% (4.5–6.6)	94.5% (93.4–95.6)
2019–2020	10.5% (9.9–11.1)	3.9% (2.7–5)	96.1% (95–97.3)
2021	12.3% (11.9–13.5)	3.7% (2.2–5.2)	96.3% (94.8–97.8)

AUD = past-year alcohol use disorder, MDE = past-year major depressive episode. AUD treatment could have been received at various locations, including hospitals, rehabilitation facilities, mental health centers, private physician’s offices, substance abuse-related emergency rooms, or self-help groups.

### Prevalence ratios for AUD and AUD treatment

Using Poisson regression models with log links, we observed that pregnant and parenting women displayed lower risk of having AUD in the past year relative to non-pregnant/non-parenting women in a bivariate model (PR = 0.48, 95% CI:0.41–0.57 and PR = 0.5, 95% CI:0.48–0.54, respectively; see Table 3). Further, the bivariate analysis showed the excess risk for past-year AUD associated with income (lowest vs. highest income, PR = 1.19, 95% CI:1.1–1.29) and history of arrests (PR = 2.93, 95% CI:2.67–3.21). The trends remained in the fully adjusted models. Additionally, older age (more than 26 years old), race other than White, and residence in a small metro or non-metro area were protective against AUD after adjusting for SES and non-SES variables.

**Table 3:** Prevalence ratios (PR) to describe the risk of having AUD in the past 12 months among reproductive-age women in the NSDUH data, 2015–2021 (weighted n = 66,519,300).

	Bivariate Associations	Model 1	Model 2
	PR (95% CI)	aPR (95% CI)	aPR (95% CI)
**Parenting status**			
Not pregnant, Not Parenting	1	1	1
Pregnant	0.48 (0.41–0.57)	0.49 (0.41–0.59)	0.51 (0.43–0.61)
Not Pregnant, Parenting	0.50 (0.48–0.54)	0.61 (0.57–0.65)	0.62 (0.58–0.66)
**Age**			
18–25	1	1	1
26–34	0.72 (0.68–0.76)	0.85 (0.80–0.91)	0.84 (0.79–0.9)
35–49	0.51 (0.48–0.53)	0.62 (0.59–0.66)	0.63 (0.60–0.67)
**Race/Ethnicity**			
White	1	1	1
Black/African American	0.80 (0.74–0.86)	0.76 (0.70–0.82)	0.76 (0.70–0.83)
Hispanic	0.70 (0.65–0.75)	0.68 (0.63–0.73)	0.71 (0.66–0.77)
Other	0.75 (0.66–0.85)	0.7 (0.62–0.80)	0.70 (0.62–0.80)
**Residence area type**			
Large Metro	1	1	1
Small Metro	0.94 (0.87–1.003)	0.89 (0.84–0.95)	0.89 (0.83–0.94)
Non-Metro	0.77 (0.71–0.85)	0.71 (0.65–0.78)	0.72 (0.66–0.79)
**Arrested during past 12 months**			
Yes	2.93, (2.67–3.21)	2.94 (2.68–3.22)	3.06 (2.79–3.37)
No	1	1	1
**Year**			
2015–2106	0.67 (0.62–0.72)	0.67 (0.63–0.73)	0.67 (0.62–0.72)
2017–2018	0.65 (0.60–0.70)	0.65 (0.60–0.70)	0.65 (0.60–0.70)
2019–2020	0.83 (0.76–0.90)	0.83 (0.76–0.90)	0.83 (0.76–0.90)
2021	1	1	1
**Education**			
Less than High School	0.63 (0.56–0.70)		0.63 (0.56–0.71)
High School	0.87 (0.81–0.94)		0.79 (0.71–0.86)
Some College/Associate Degree	1.07 (1.01–1.13)		0.97 (0.91–1.04)
College Graduate	1		1
**Annual Household Income**			
Less than $20,000	1.19 (1.10–1.29)		1.26 (1.15–1.39)
$20,000-$49,999	1.09 (1.03–1.16)		1.17 (1.09–1.26)
$50,000-$74,999	1.05 (0.97–1.13)		1.07 (0.99–1.16)
$75,000+	1		1
**Health Insurance**			
Private	1		1
Medicaid/CHIP	0.89 (0.83–0.95)		0.94 (0.87–1.02)
Medicare	0.69 (0.51–0.92)		0.72 (0.53–0.98)
Other	1.04 (0.90–1.05)		1.01 (0.88–1.16)
No insurance	0.97 (0.91–1.19)		1.02 (0.93–1.13)

Using the same modeling approach, we did not observe differences in AUD treatment use based on parenting or pregnancy status (see Table 4). In both the bivariate and multivariate analyses, individuals over 26 years old had one and a half the likelihood of receiving treatment compared to those aged 18–25 years. Enrollment in Medicaid significantly increased the chances of receiving treatment when contrasted with individuals having private insurance (PR = 2.62, 95% CI: 1.97–3.47), and this relationship remained robust after adjusting for confounding factors (aPR = 1.68, 95%CI: 1.23–2.28). Similarly, lower education (some college/associate degree) was significantly associated with a higher prevalence of treatment when compared to college graduates, and the association remained after controlling for income, history of arrests, age, and the type of health insurance. History of arrests displayed the strongest association with treatment receipt, maintaining its significance after adjusting for confounders (aPR = 5.37, 95%CI: 3.92–7.34).

**Table 4:** Prevalence ratios for AUD treatment among reproductive age women with AUD.

	Bivariate associations	Model 1	Model 2
	PR (95% CI)	aPR (95% CI)	aPR (95% CI)
**Parenting status**			
Not pregnant, Not Parenting	1.00	1.00	1.00
Pregnant	1.24 (0.59–2.61)	1.07 (0.55–2.47)	0.93 (0.47–1.86)
Not Pregnant, Parenting	1.19 (0.88–1.61)	0.99 (0.71–1.37)	0.90 (0.67–1.21)
**Age**			
18–25	1	1	1
26–34	1.48 (1.04–2.09)	1.51 (1.09–2.11)	1.59 (1.13–2.23)
35–49	1.6 (1.19–2.14)	1.7 (1.26–2.31)	1.91 (1.38–2.64)
**Race/Ethnicity**			
White	1	1	1
Black/African American	0.87 (0.58–1.31)	0.81 (0.53–1.24)	0.66 (0.43–1.02)
Hispanic	0.88 (0.61–1.25)	0.89 (0.62–1.28)	0.76 (0.50–1.14)
Other	0.65 (0.46–0.93)	0.65 (0.46–0.93)	0.6 (0.40–0.86)
**Residence area type**			
Large Metro	1	1	1
Small Metro	1.33 (1.04–1.70)	1.27 (1.01–1.59)	1.17 (0.90–1.52)
Non-Metro	1.43 (0.94–2.18)	1.23 (0.79–1.93)	1.03 (0.66–1.61)
**Arrested during past 12 months**			
No	1	1	1
Yes	7.31 (5.56–9.60)	7.16 (5.40–9.49)	5.37 (3.92–7.34)
**Year**			
2015–2106	1.66 (0.83–3.33)	1.2 (0.77–1.86)	1.17 (0.75–1.81)
2017–2018	2.07 (0.91–4.69)	1.36 (0.86–2.15)	1.36 (0.86–2.16)
2019–2020	0.83 (0.32–2.15)	0.99 (0.61–1.59)	0.98 (0.61–1.59)
2021	1	1	1
**Education**			
Less than High School	2.88 (1.92–4.32)		1.54 (0.91–2.6)
High School	1.95 (1.30–2.92)		1.43 (0.91–2.25)
Some College/Associate Degree	1.82 (1.30–2.54)		1.56 (1.06–2.30)
College Graduate	1		1
**Annual Household Income**			
Less than $20,000	2.29 (1.67–3.14)		1.33 (0.87–2.02)
$20,000-$49,999	1.45 (1.04–2.03)		1 (0.65–1.55)
$50,000-$74,999	1.33 (0.96–1.83)		1.14 (0.81–1.62)
$75,000+	1		1
**Health Insurance**			
Private	1		1
Medicaid/CHIP	2.62 (1.97–3.47)		1.68 (1.23–2.28)
Medicare	1.59 (0.66–3.83)		0.77 (0.34–1.74)
Other	1.37 (0.88–2.14)		1.34 (0.83–2.14)
No insurance	1.97 (1.31–2.96)		1.38 (0.91–2.09)

Treatment could have been received at various locations, including hospitals, rehabilitation facilities, mental health centers, private physician’s offices, substance abuse-related emergency rooms, or self-help groups.

### Barriers to treatment

**Fig 1:**
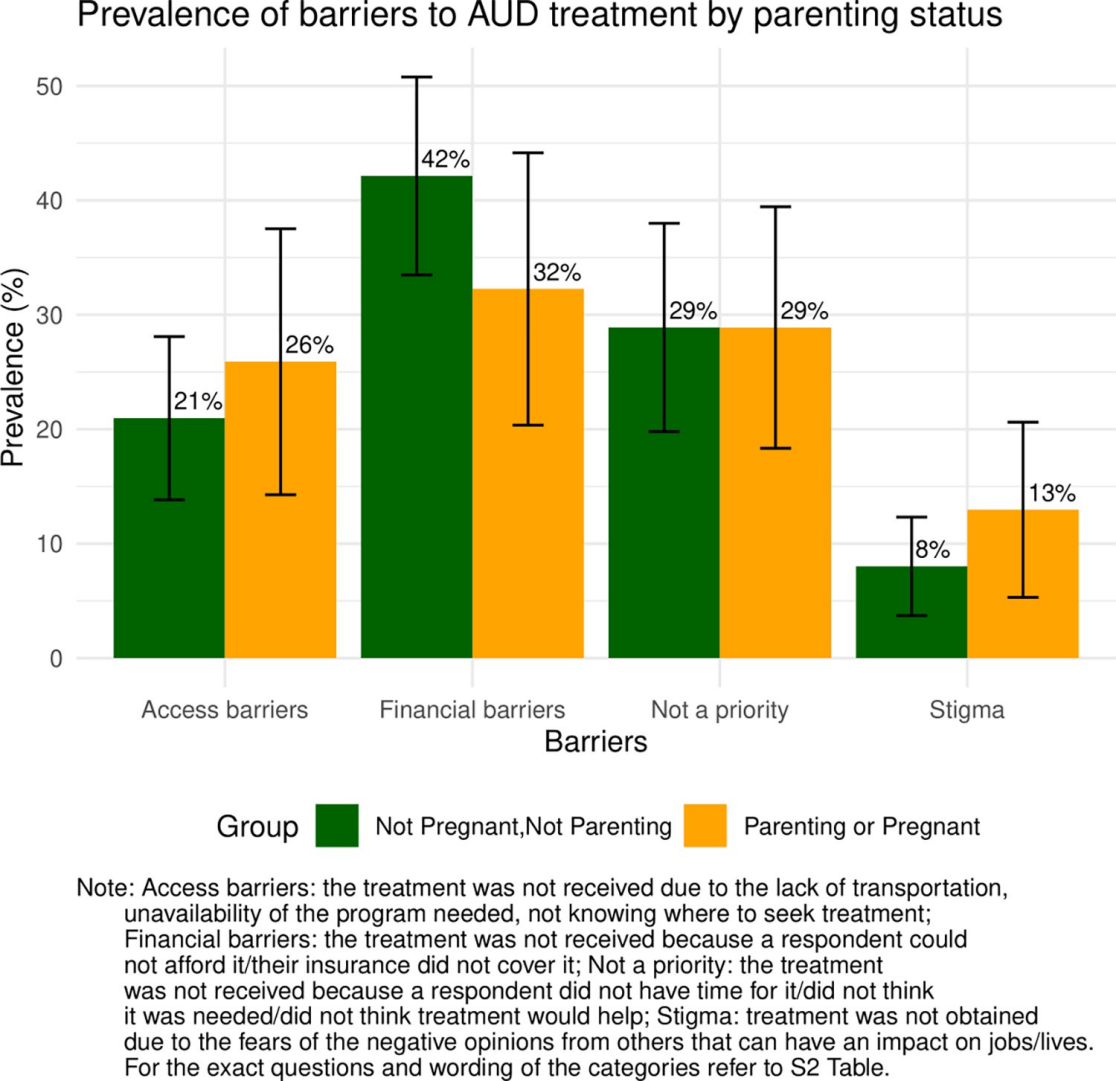
Prevalence of reported barriers to AUD treatment by parenting status.

Due to limitations in sample size, we combined pregnant and parenting women into a single category for barrier analysis (total n = 559, weighted N = 6,033,701). Among those with a treatment need but not receiving it, financial barriers emerged as the most frequently reported obstacle, endorsed by 42% (95%CI: 33.5–51%) of non-pregnant, non-parenting women and 32% (95%CI: 20.4–44.2%) of pregnant/parenting individuals. The second-ranked barrier was the treatment not being a priority, with nearly 30% of respondents from both groups identifying it. Access barriers and stigma were more prevalent among the parenting/pregnant group, with rates of 26% and 13%, respectively, compared to 21% and 8% in the other category (Fig 1), although these estimates were not precise.

## Discussion

The present study aimed to update the prevalence and treatment patterns of past-year AUD among reproductive-age women and explore the barriers to treatment within the group. The findings shed light on important factors associated with AUD and highlight the need for targeted interventions to address the unique challenges faced by reproductive-age women in the US. By looking at a nationally representative sample of reproductive-age women, we found that the prevalence of past twelve months AUD varied across those who reported being pregnant, parenting or non-pregnant non-parenting at the time of data collection. Approximately 6.3 percent of pregnant women and 6.6 percent of parenting women had AUD in the past year, while the prevalence among non-pregnant/non-parenting women was higher at 13 percent. These findings suggest that pregnancy and parenting may be associated with a lower risk of AUD consistent with previous research [16]. The identified association between higher education and an elevated risk of AUD in reproductive-age females is consistent with findings in studies by Schnuerer et al. and others [17–19]. Additionally, our observations regarding metropolitan residences and an increased risk of AUD align with patterns highlighted in NSDUH annual reports [20, 21]. Collectively, while these trends may seem counterintuitive, they emphasize the importance of addressing alcohol misuse among females, irrespective of their socioeconomic status.

The finding that women who are not pregnant/not parenting are at a higher risk of AUD aligns with recent work by Adams et al. (2023), who identified a time trend of increasing binge drinking and AUD among child-free women of reproductive age in the US (OR = 1.73, 95%CI: 1.42–2.12, in 2018–2019 relative to 1993–1997) [22]. Additionally, there was an inverse association between the transition to parenthood and excessive drinking (the ORs range for binge drinking among 18-24-year-old women without children compared to those who had children was 1.22–1.55) [22]. The increased risk of AUD in the non-pregnant, non-parenting group also extends to inadvertent alcohol exposure during the early stages of an unplanned pregnancy. Considering that these women are in their primary child-bearing years along with the fact that approximately half of the pregnancies are unintended [23], there is a heightened likelihood of fetal alcohol exposure during sensitive periods of pregnancy [24]. These findings underscore the need to address excessive drinking among all women, and particularly, to not overlook the issue of binge drinking among the expanding group of women without children.

The prevalence of the past-year AUD among women of childbearing age experienced a notable increase around 2020, rising from approximately 8 percent in the preceding years to 10.5 percent. While the NSDUH methodology advises against direct comparisons of the 2020–2021 data to previous years, it is important to acknowledge that this shift likely stems from the profound impact of COVID-19. The Johns Hopkins-University of Maryland-Baltimore survey revealed that 60.1% of participants reported heightened alcohol consumption after March 1, 2020. This coincided with a remarkable surge in online alcohol sales by 234% and a 21% increase in in-store sales compared to the previous year [25].

Among reproductive-age women who reported having AUD in the past 12 months, less than 6 percent received treatment regardless of their parenting/pregnancy status. Even though pregnant individuals are considered a priority group [26] and should be prioritized in access to treatment, our findings did not indicate such a trend. The findings may be partially attributed to the fact that certain medications used for AUD treatment [27] are not recommended for pregnant individuals [28]; further investigation into this aspect was hindered by data limitations. When considering all available treatment options, there was no clear difference between the categories of women with AUD in the prevalence ratios for access to AUD treatment after adjusting for confounding. Our results align with previous reports drawing attention to unmet substance use treatment need in pregnant individuals [10, 29–31]. In the context of the ongoing opioid epidemic, which has witnessed a nearly threefold increase in drug overdose mortality during pregnancy [32], it is vital to acknowledge the frequent co-occurrence of AUD with other substance use [33, 34]. Specifically, 38% of pregnant individuals report using at least one other substance alongside alcohol [34]. Neuroimaging research confirms that exposure to multiple substances during pregnancy has enduring effects on offspring’s brain structure and cognitive function [35]. This underscores the urgent need to address untreated addictions in reproductive-age women. Despite the recognition of this issue and efforts to emphasize the importance of providing adequate treatment and support for pregnant women [10, 36], our study did not find an improvement in access to treatment over the years under investigation.

History of arrests displayed the strongest association with both AUD and AUD treatment. The link between alcohol misuse and criminal behavior may be bidirectional, with AUD increasing the likelihood of engaging in criminal activities [37] and involvement with the criminal justice system increasing the risk of substance use disorders [38]. Women with a history of arrests may have encountered legal consequences related to their substance use, such as court-mandated treatment or probation requirements [39–41]. Thus, exposure to the criminal justice system may explain why a history of arrests is associated with higher treatment engagement [42]. In cases when women have been separated from their children due to criminal involvement, the desire to be reunited can act as a motivating factor for seeking treatment, particularly when mandated by Child Protective Services [43].

The most frequently reported barrier to treatment was financial barriers, suggesting that addressing the cost of treatment and providing financial support may be crucial in increasing treatment utilization among reproductive-age women. Low prioritization was the second prevalent barrier in our study population. A recent scoping review demonstrated that the prevalence of maternal alcohol misuse increases with a child’s age, peaking around the child’s age of 12–14 [44]. Childhood and adolescence are critical periods marked by substantial transformations in body, brain, and behavior [45]. Interactions and exposures during this time play a pivotal role in shaping pathways to adulthood and laying the foundation for future health and cognitive outcomes. Given the potentially traumatizing impact of parental alcohol misuse, there is a crucial need for enhanced prevention efforts, the identification of at-risk children, and the implementation of family-based interventions [44]. Furthermore, there is a call for increased awareness regarding PAE’s potential harms and highlighting the benefits of discontinuing alcohol consumption at any stage of pregnancy [46]. Increasing knowledge about the consequences of alcohol use during pregnancy can empower individuals, including healthcare providers, policymakers, and the general public, to prioritize the treatment and support of pregnant individuals with AUD [47]. Education efforts should not only target pregnant individuals themselves but should also extend to their support networks, including partners, family members, and healthcare providers [48]. The finding also underscores the need to continually address the stigma surrounding AUD treatment during pregnancy and parenting, as it remains more noticeable in these groups of women.

This study provides a comprehensive analysis of AUD trends and its treatment among reproductive-age women from 2015 to 2021, with a specific focus on those who reported being pregnant and/or parenting. The research utilizes a nationally representative sample and applies the most recent diagnostic criteria from the DSM-5 to identify individuals with AUD. The study findings should be generalizable to non-institutionalized women of child-bearing age residing in the US. It is important to acknowledge some limitations of the current study. First, NSDUH relies on self-reported measures for alcohol use. If individuals did not remember or were unwilling to disclose the accurate history of use, recall or social desirability bias could have led to under-estimates of AUD. To mitigate this issue, the NSDUH survey uses Audio Computer-Assisted Self-Interview Software to gather responses to sensitive questions. Second, due to the limitations of the survey items, we were not able to evaluate the risk of AUD during pregnancy specifically but the risk of past-year AUD in those people who also reported pregnancy at the time of the data collection. While AUD is known to have a prolonged duration and recovery period [49], potentially mitigating bias, it is important to acknowledge the possibility of some degree of misclassification bias in the obtained results. Third, because of the COVID-19 pandemic, the NSDUH data collection starting in 2020 underwent significant changes. Specifically, no data were collected between mid-March through September. Beginning in October, the NSDUH used web rather than in-person data collection. This may bias our results if the 2020–2021 sample of respondents differed or if they reported past-year alcohol use differently compared to respondents in previous years. To address this concern, we include a sensitivity analysis limited to 2015–2019 data, and the results were consistent with the full study findings. Due to the sample size constraints, we were only able to identify barriers in the combined category of parenting and pregnant women. These two groups may face different barriers to treatment, but our analysis could not explore the unique challenges each group may encounter. Further, the limited sample size prevented an in-depth exploration of barriers beyond the predetermined four categories in the available data, which would have enhanced the analysis.

Future research should aim to utilize larger and more diverse samples of pregnant and parenting women to validate and extend the current findings. Longitudinal studies are necessary to explore causal risk factors for AUD in reproductive-age women. Expanding the scope to include polysubstance use, along with an exploration of co-occurring mental health issues, is essential for a more holistic understanding of the risks associated with AUD and its treatment patterns. Additionally, qualitative research methods could provide more in-depth insights into the barriers to treatment and the experiences of pregnant and parenting women with AUD.

## Supporting information

S1 Table DSM-5 past-year AUD variable construction. (DOCX)

S2 Table Barriers questions and groupings. (DOCX)

S3 Table Sensitivity analysis for prevalence ratios of having past-year AUD among reproductive age women. (DOCX)

S4 Table Sensitivity analysis for prevalence ratios of AUD treatment among reproductive age women with AUD. (DOCX)

S5 Table Regression outputs produced during the analysis. (DOCX)
