# Supplementary material for: Unmet need for alcohol use disorder treatment in reproductive-age females, with emphasis on pregnant and parenting populations in the United States: Findings from NSDUH 2015–2021
Source: PLoS One. 2024 Apr 9;19(4):e0301810. doi: 10.1371/journal.pone.0301810 (PMC11003670; doi:10.1371/journal.pone.0301810)
Supplement: S1 Table — (DOCX) [file pone.0301810.s001.docx]

| **S1 Table.** DSM-5 past-year AUD variable construction | | |
| --- | --- | --- |
| **DSM-5 AUD Criteria** | **NSDUH Variable** | **Survey Question** |
| 1. Alcohol is often taken in larger amount or over a longer period than was intended. | ALCKPLMT | Were you able to keep to the limits you set, or did you often drink more than you intended to? |
| 1. There was a persistent desire or unsuccessful efforts to cut down or control alcohol use. | ALCCUTEV | During the past 12 months, were you able to cut down or stop drinking alcohol every time you wanted to or tried to? |
| 1. A great deal of time is spent in activities necessary to obtain alcohol, use alcohol, or recover from its effects. | ALCLOTTM  *or* ALCGOTVR | During the past 12 months, was there a month or more when you spent a lot of your time getting or drinking alcohol?  *or*  During the past 12 months, was there a month or more when you spent a lot of time getting over the effects of the alcohol you drank? |
| 1. Craving, or a strong desire of urge to use alcohol. | Not assessed | Not assessed |
| 1. Recurrent alcohol use resulting in a failure to fulfill major role obligations at work, school, or home. | ALCSERPB | During the past 12 months, did drinking alcohol cause you to have serious problems like this either at home, work, or school? |
| 1. Continued alcohol use despite having persistent or recurrent social or interpersonal problems caused or exacerbated by the effects of opioids. | ALCFMCTD | Did you continue to drink alcohol even though you thought your drinking caused problems with family or friends? |
| 1. Important social, occupational, or recreational activities are given up or reduced because of alcohol use. | ALCLSACT | During the past 12 months, did drinking alcohol cause you to give up or spend less time doing these types of important activities? |
| 1. Recurrent alcohol use in situations in which it is physically hazardous. | ALCPDANG | During the past 12 months, did you regularly drink alcohol and then do something where being drunk might have put you in physical danger? |
| 1. Continued alcohol use despite knowledge of having a persistent or recurrent physical or physiological problem that is likely to have been caused or exacerbated by the substance. | ALCEMCTD *or*  ALCPHCTD | Did you continue to drink alcohol even though you thought your drinking caused problems with family or friends?   *or*  Did you continue to drink alcohol even though you thought drinking was causing you to have physical problems? |
| 1. Tolerance, as defined by either of the following: a) A need for markedly increased amounts of alcohol to achieve intoxication or desired effect: b) A markedly diminished effect with continued use of the same amount of alcohol. | ALCNDMOR  *or*  ALCLSEFX | During the past 12 months, did you need to drink more alcohol than you used to in order to get the effect you wanted?   *or*  During the past 12 months, did you notice that drinking the same amount of alcohol had less effect on you than it used to? |
| 1. Withdrawal, as manifested by either of the following: a) The characteristic withdrawal syndrome for alcohol; b) Alcohol (or a closely related substance, such as a benzodiazepine) is taken to relieve or avoid withdrawal symptoms. | ALCWD2SX | Please look at the symptoms listed below. During the past 12 months, did you have 2 or more of these symptoms after you cut back or stopped drinking alcohol?   - Sweating or feeling that your heart was beating fast - Having your hands tremble - Having trouble sleeping - Vomiting or feeling nauseous - Seeing, hearing, or feeling things that weren’t really there - Feeling like you couldn’t sit still - Feeling anxious - Having seizures or fits |
