# Supplementary material for: Unmet need for alcohol use disorder treatment in reproductive-age females, with emphasis on pregnant and parenting populations in the United States: Findings from NSDUH 2015–2021
Source: PLoS One. 2024 Apr 9;19(4):e0301810. doi: 10.1371/journal.pone.0301810 (PMC11003670; doi:10.1371/journal.pone.0301810)
Supplement: S2 Table — (DOCX) [file pone.0301810.s002.docx]

| **S2 Table.** Barriers questions and groupings | | |
| --- | --- | --- |
| **NSDUH Variable** | **NSDUH Question** | **Grouping** |
| NDTXNOCOV, NDMRNOCOV | 1. You had no health care coverage, and you couldn’t afford the cost. | Financial barriers |
| NDTXNOTPY, NDMRNOTPY | 2.  You did have health care coverage, but it didn’t cover treatment or didn’t cover the full cost. | Financial barriers |
| NDTXTSPHR, NDMRTSPHR | 3. You had no transportation to a program, or the programs were too far away, or the hours were not convenient. | Access barriers |
| NDTXWANTD, NDMRWANTD | 4. You didn’t find a program that offered the type of treatment or counseling you wanted. | Access barriers |
| NDTXNSTOP, NDMRNSTOP | 5. You were not ready to stop using. | Not a priority |
| NDTXPFULL, NDMRPFULL | 6. There were no openings in the programs. | Access barriers |
| NDTXDKWHR, NDMRDKWHR | 7. You did not know where to go to get treatment. | Access barriers |
| NDTXNBRNG, NDMRNBRNG | 8. You were concerned that getting treatment or counseling might cause your neighbors or community to have a negative opinion of you. | Stigma |
| NDTXJOBNG, NDMRJOBNG | 9. You were concerned that getting treatment or counseling might have a negative effect on your job. | Stigma |
| NDTXNONED, NDMRNONED | 10.You didn't think you needed treatment at the time. | Not a priority |
| NDTXHANDL, NDMRHANDL | 11. You thought you could handle the problem without treatment. | Not a priority |
| NDTXNOHLP, NDMRNOHLP | 12. You didn't think treatment would help. | Not a priority |
| NDTXNTIME, NDMRNTIME | 13. You didn't have time (because of job, childcare, or other commitments). | Not a priority |
| NDTXFNDOU, NDMRFNDOU | 14. You didn't want others to find out that you needed treatment. | Stigma |
