# Supplementary material for: Unmet need for alcohol use disorder treatment in reproductive-age females, with emphasis on pregnant and parenting populations in the United States: Findings from NSDUH 2015–2021
Source: PLoS One. 2024 Apr 9;19(4):e0301810. doi: 10.1371/journal.pone.0301810 (PMC11003670; doi:10.1371/journal.pone.0301810)
Supplement: S3 Table — (DOCX) [file pone.0301810.s003.docx]

| **S3_Table.** Sensitivity analysis for prevalence ratios of having past-year AUD among reproductive-age women. | | |
| --- | --- | --- |
|  | **Sensitivity analysis**  **(2015-2019)**  **Adjusted PR**  (weighted N= 48,236,346) | **Sensitivity analysis  (2020-2021)**  **Adjusted PR**  (weighted N= 18,282,954) |
| **Parenting status** |  |  |
| Not pregnant, Not Parenting | 1 | 1 |
| Pregnant | 0.48 (0.39-0.58) | 0.57 (0.41-0.80) |
| Not Pregnant, Parenting | 0.57 (0.54-0.61) | 0.72 (0.63-0.82) |
| **Age** |  |  |
| 18-25 | 1 | 1 |
| 26-34 | 0.78 (0.73-0.84) | 0.97 (0.85-1.11) |
| 35-49 | 0.56 (0.52-0.60) | 0.78 (0.70-0.88) |
| **Race/Ethnicity** |  |  |
| White | 1 | 1 |
| Black/African American | 0.77 (0.7-0.84) | 0.75 (0.63-0.9) |
| Hispanic | 0.75 (0.69-0.81) | 0.66 (0.55-0.79) |
| Other | 0.72 (0.64-0.81) | 0.68 (0.54-0.86) |
| **Residence area type** |  |  |
| Large Metro | 1 | 1 |
| Small Metro | 0.88 (0.82-0.94) | 0.9 (0.81-1.02) |
| Non-Metro | 0.71 (0.65-0.78) | 0.73 (0.61-0.87) |
| **Arrested during past 12 months** |  |  |
| Yes | 3.18 (2.87-3.52) | 2.72 (2.06-3.59) |
| No | 1 | 1 |
| **Year** |  |  |
| 2015 | 1.00 |  |
| 2016 | 0.98 (0.89-1.08) | - |
| 2017 | 0.95 (0.88-1.03) | - |
| 2018 | 0.96 (0.88-1.04) | - |
| 2019 | 0.99 (0.90-1.08) | - |
| 2020 |  | 1.01 (0.89-1.10) |
| 2021 |  | 1.00 |
| **Education** |  |  |
| Less than High School | 0.64 (0.58-0.72) | 0.62 (0.46-0.82) |
| High School | 0.84 (0.75-0.93) | 0.72 (0.58-0.88) |
| Some College/Associate Degree | 0.97(0.90-1.05) | 0.99 (0.86-1.15) |
| College Graduate | 1 | 1 |
| **Annual Household Income** |  |  |
| Less than $20,000 | 1.19 (1.08-1.32) | 1.38 (1.14-1.68) |
| $20,000-$49,999 | 1.1 (1.02-1.19) | 1.32 (1.17-1.49) |
| $50,000-$74,999 | 1.03 (0.95-1.11) | 1.15 (0.96-1.38) |
| $75,000+ | 1 | 1 |
| **Health Insurance** |  |  |
| Private | 1 | 1 |
| Medicaid/CHIP | 0.99 (0.91-1.08) | 0.84 (0.70-1) |
| Medicare | 0.71 (0.51-1) | 0.7 (0.40-1.21) |
| Other | 0.97 (0.84-1.12) | 1.09 (0.80-1.49) |
| No insurance | 0.99 (0.89-1.09) | 1.08 (0.90-1.32) |
