# Supplementary material for: Unmet need for alcohol use disorder treatment in reproductive-age females, with emphasis on pregnant and parenting populations in the United States: Findings from NSDUH 2015–2021
Source: PLoS One. 2024 Apr 9;19(4):e0301810. doi: 10.1371/journal.pone.0301810 (PMC11003670; doi:10.1371/journal.pone.0301810)
Supplement: S4 Table — (DOCX) [file pone.0301810.s004.docx]

| **S4_Table.** Sensitivity analysis for prevalence ratios of AUD treatment among reproductive-age women with AUD. | | |
| --- | --- | --- |
|  | **2015-2019**  (Weighted N = 4,002,147) | **2020-2021**  (Weighted N = 2,314,670) |
| **Parenting status** |  |  |
| Not pregnant, Not Parenting | 1 | 1 |
| Pregnant | 0.77 (0.35-1.71) | 1.61 (0.45-5.69) |
| Not Pregnant, Parenting | 1 (0.74-1.35) | 0.72 (0.38-1.39) |
| **Age** |  |  |
| 18-25 | 1 | 1 |
| 26-34 | 1.58 (1.16-2.14) | 1.54 (0.64-3.73) |
| 35-49 | 1.92 (1.43-2.58) | 1.94 (0.79-4.78) |
| **Race/Ethnicity** |  |  |
| White | 1 | 1 |
| Black/African American | 0.67 (0.43-1.04) | 0.72 (0.28-1.88) |
| Hispanic | 0.78 (0.48-1.26) | 0.8 (0.34-1.91) |
| Other | 0.57 (0.34-0.94) | 0.72 (0.3-1.71) |
| **Residence area type** |  |  |
| Large Metro | 1 | 1 |
| Small Metro | 1.05 (0.79-1.39) | 1.85 (1.07-3.19) |
| Non-Metro | 0.82 (0.54-1.25) | 1.94 (0.79-4.75) |
| **Arrested during past 12 months** |  |  |
| No | 1 | 1 |
| Yes | 5.16 (3.66-7.28) | 5.27 (2.58-10.77) |
| **Year** |  |  |
| 2015 | 1.00 |  |
| 2106 | 1.44 (0.84-2.45) | - |
| 2017 | 1.36 (0.81-2.27) | - |
| 2018 | 1.47(0.95-2.27) | - |
| 2019 | 1.37 (0.91-2.05) | - |
| 2020 | - | 1.00 |
| 2021 | - | 1.42 (0.72-2.78) |
| **Education** |  |  |
| Less than High School | 1.58 (1.03-2.43) | 1.09 (0.23-5.23) |
| High School | 1.31 (0.89-1.95) | 1.37 (0.43-4.35) |
| Some College/Associate Degree | 1.61 (1.14-2.28) | 1.18 (0.39-3.63) |
| College Graduate | 1 | 1 |
| **Annual Household Income** |  |  |
| Less than $20,000 | 0.98 (0.66-1.47) | 3.98 (1.54-10.27) |
| $20,000-$49,999 | 0.82 (0.54-1.25) | 2.19 (0.66-7.22) |
| $50,000-$74,999 | 1.13 (0.77-1.64) | 1.24 (0.38-4.03) |
| $75,000+ | 1 | 1 |
| **Health Insurance** |  |  |
| Private | 1 | 1 |
| Medicaid/CHIP | 1.66 (0.23-2.28) | 1.59 (0.85-2.97) |
| Medicare | 0.72 (1.17-2.37) | 0.84 (0.23-3.02) |
| Other | 1.41 (0.85-2.34) | 1.43 (0.39-5.26) |
| No insurance | 1.33 (0.88-2.02) | 1.59 (0.73-3.5) |
