# Supplementary material for: Unmet need for alcohol use disorder treatment in reproductive-age females, with emphasis on pregnant and parenting populations in the United States: Findings from NSDUH 2015–2021
Source: PLoS One. 2024 Apr 9;19(4):e0301810. doi: 10.1371/journal.pone.0301810 (PMC11003670; doi:10.1371/journal.pone.0301810)

Supplement materials: regression outputs (Notes: exp = exponentiated)

**PR OF PAST-YEAR AUD**

**Bivariate models**

**Parenting status**

| Variable | *b** | *SE* | *t* | *p* | exp B | exp CI_lower | exp CI_upper |
| --- | --- | --- | --- | --- | --- | --- | --- |
| (Intercept) | -2.04 | 0.02 | -116.73 | < .001*** | 0.13 | 0.13 | 0.13 |
| Not pregnant, Parenting | -0.68 | 0.03 | -23.18 | < .001*** | 0.50 | 0.48 | 0.54 |
| Pregnant | -0.73 | 0.08 | -8.68 | < .001*** | 0.48 | 0.41 | 0.57 |

**Age**

| Variable | *b** | *SE* | *t* | *p* | exp B | exp CI_lower | exp CI_upper |
| --- | --- | --- | --- | --- | --- | --- | --- |
| (Intercept) | -1.98 | 0.02 | -131.63 | < .001*** | 0.14 | 0.13 | 0.14 |
| Age: 26-34 | -0.33 | 0.03 | -11.17 | < .001*** | 0.72 | 0.68 | 0.76 |
| Age: 35-49 | -0.68 | 0.03 | -24.42 | < .001*** | 0.50 | 0.48 | 0.53 |

**Race**

| Variable | *b** | *SE* | *t* | *p* | exp B | exp CI_lower | exp CI_upper |
| --- | --- | --- | --- | --- | --- | --- | --- |
| (Intercept) | -2.23 | 0.02 | -132.07 | < .001*** | 0.11 | 0.10 | 0.11 |
| RaceBlack | -0.22 | 0.04 | -5.78 | < .001*** | 0.80 | 0.74 | 0.86 |
| RaceHispanic | -0.36 | 0.04 | -9.87 | < .001*** | 0.70 | 0.65 | 0.75 |
| RaceOther | -0.29 | 0.06 | -4.59 | < .001*** | 0.75 | 0.66 | 0.85 |

**Education**

| Variable | *b** | *SE* | *t* | *p* | exp B | exp CI_lower | exp CI_upper |
| --- | --- | --- | --- | --- | --- | --- | --- |
| (Intercept) | -2.30 | 0.02 | -95.56 | < .001*** | 0.10 | 0.10 | 0.10 |
| <High School | -0.46 | 0.06 | -8.27 | < .001*** | 0.63 | 0.56 | 0.70 |
| High School | -0.14 | 0.04 | -3.59 | .001*** | 0.87 | 0.81 | 0.94 |
| Some College | 0.06 | 0.03 | 2.23 | .031* | 1.07 | 1.01 | 1.13 |

**Income**

| Variable | *b** | *SE* | *t* | *p* | exp B | exp CI_lower | exp CI_upper |
| --- | --- | --- | --- | --- | --- | --- | --- |
| (Intercept) | -2.41 | 0.02 | -115.15 | < .001*** | 0.09 | 0.09 | 0.09 |
| < $20,000 | 0.17 | 0.04 | 4.43 | < .001*** | 1.19 | 1.10 | 1.29 |
| $20,000 - $49,999 | 0.09 | 0.03 | 3.08 | .003** | 1.09 | 1.03 | 1.16 |
| $50,000 - $74,999 | 0.04 | 0.04 | 1.15 | .257 | 1.04 | 0.97 | 1.13 |

**Insurance**

| Variable | *b** | *SE* | *t* | *p* | exp B | exp CI_lower | exp CI_upper |
| --- | --- | --- | --- | --- | --- | --- | --- |
| (Intercept) | -2.32 | 0.01 | -157.99 | < .001*** | 0.10 | 0.10 | 0.10 |
| Medicaid/CHIP | -0.12 | 0.04 | -3.44 | .001** | 0.89 | 0.82 | 0.95 |
| Medicare | -0.38 | 0.15 | -2.57 | .013* | 0.69 | 0.51 | 0.92 |
| No insurance | -0.03 | 0.04 | -0.69 | .497 | 0.97 | 0.90 | 1.05 |
| Other | 0.04 | 0.07 | 0.56 | .576 | 1.04 | 0.91 | 1.19 |

**Metro**

| Variable | *b** | *SE* | *t* | *p* | exp B | exp CI_lower | exp CI_upper |
| --- | --- | --- | --- | --- | --- | --- | --- |
| (Intercept) | -2.30 | 0.02 | -112.05 | < .001*** | 0.10 | 0.10 | 0.10 |
| Non-Metro | -0.26 | 0.04 | -5.78 | < .001*** | 0.77 | 0.71 | 0.84 |
| Small Metro | -0.06 | 0.03 | -1.93 | .060 | 0.94 | 0.89 | 1.00 |

**Year**

| Variable | *b** | *SE* | *t* | *p* | exp B | exp CI_lower | exp CI_upper |
| --- | --- | --- | --- | --- | --- | --- | --- |
| (Intercept) | -2.06 | 0.03 | -67.25 | < .001*** | 0.13 | 0.12 | 0.14 |
| 2015-2016 | -0.40 | 0.04 | -11.01 | < .001*** | 0.67 | 0.62 | 0.72 |
| 2017-2018 | -0.44 | 0.04 | -11.74 | < .001*** | 0.65 | 0.60 | 0.70 |
| 2019-2020 | -0.19 | 0.04 | -4.81 | < .001*** | 0.83 | 0.76 | 0.90 |

**PR of AUD, the output from a model adjusted for non-SES variables**

| Variable | *b** | *SE* | *t* | *p* | exp B | exp CI_lower | exp CI_upper |
| --- | --- | --- | --- | --- | --- | --- | --- |
| (Intercept) | -1.43 | 0.04 | -35.75 | < .001*** | 0.24 | 0.22 | 0.26 |
| Age: 26-34 | -0.16 | 0.03 | -5.02 | < .001*** | 0.85 | 0.80 | 0.91 |
| Age: 35-49 | -0.47 | 0.03 | -17.21 | < .001*** | 0.62 | 0.59 | 0.66 |
| Race: Black | -0.27 | 0.04 | -7.00 | < .001*** | 0.76 | 0.70 | 0.82 |
| Race: Hispanic | -0.38 | 0.04 | -10.55 | < .001*** | 0.68 | 0.63 | 0.73 |
| Race: Other | -0.35 | 0.06 | -5.69 | < .001*** | 0.70 | 0.62 | 0.80 |
| Metro: Non-Metro | -0.35 | 0.05 | -7.64 | < .001*** | 0.71 | 0.64 | 0.78 |
| Metro: Small Metro | -0.12 | 0.03 | -3.82 | < .001*** | 0.89 | 0.84 | 0.95 |
| Year: 2015-2016 | -0.39 | 0.04 | -10.86 | < .001*** | 0.67 | 0.63 | 0.73 |
| Year: 2017-2018 | -0.43 | 0.04 | -11.23 | < .001*** | 0.65 | 0.60 | 0.70 |
| Year: 2019-2020 | -0.19 | 0.04 | -4.87 | < .001*** | 0.83 | 0.76 | 0.89 |
| Arrested during the past 12m | 1.08 | 0.05 | 23.74 | < .001*** | 2.94 | 2.68 | 3.22 |
| Not pregnant, Parenting | -0.50 | 0.03 | -16.45 | < .001*** | 0.61 | 0.57 | 0.65 |
| Pregnant | -0.72 | 0.09 | -8.12 | < .001*** | 0.49 | 0.41 | 0.58 |

**PR for AUD fully adjusted model output**

| Variable | *b** | *SE* | *t* | *p* | exp B | exp CI_lower | exp CI_upper |
| --- | --- | --- | --- | --- | --- | --- | --- |
| (Intercept) | -1.44 | 0.05 | -31.94 | < .001*** | 0.24 | 0.22 | 0.26 |
| Insurance: Medicaid/CHIP | -0.06 | 0.04 | -1.53 | .137 | 0.94 | 0.87 | 1.02 |
| Insurance: Medicare | -0.33 | 0.15 | -2.18 | .039* | 0.72 | 0.53 | 0.98 |
| Insurance: No insurance | 0.02 | 0.05 | 0.51 | .615 | 1.02 | 0.93 | 1.13 |
| Insurance: Other | 0.01 | 0.07 | 0.18 | .855 | 1.01 | 0.88 | 1.16 |
| Age:26-34 | -0.18 | 0.03 | -5.42 | < .001*** | 0.84 | 0.79 | 0.90 |
| Age: 35-49 | -0.46 | 0.03 | -16.92 | < .001*** | 0.63 | 0.60 | 0.67 |
| Race: Black | -0.27 | 0.04 | -6.81 | < .001*** | 0.76 | 0.70 | 0.83 |
| Race: Hispanic | -0.34 | 0.04 | -8.92 | < .001*** | 0.71 | 0.66 | 0.77 |
| Race: Other | -0.35 | 0.06 | -5.67 | < .001*** | 0.70 | 0.62 | 0.80 |
| Metro: Non-Metro | -0.33 | 0.04 | -7.48 | < .001*** | 0.72 | 0.66 | 0.79 |
| Metro: Small Metro | -0.12 | 0.03 | -3.95 | .001*** | 0.89 | 0.83 | 0.94 |
| Year: 2015-2016 | -0.40 | 0.04 | -11.10 | < .001*** | 0.67 | 0.62 | 0.72 |
| Year: 2017-2018 | -0.44 | 0.04 | -11.54 | < .001*** | 0.65 | 0.60 | 0.70 |
| Year: 2019-2020 | -0.19 | 0.04 | -4.83 | < .001*** | 0.83 | 0.76 | 0.90 |
| Not pregnant, Parenting | -0.48 | 0.03 | -15.46 | < .001*** | 0.62 | 0.58 | 0.66 |
| Pregnant | -0.68 | 0.09 | -7.70 | < .001*** | 0.51 | 0.42 | 0.61 |
| Education: < High School | -0.47 | 0.06 | -8.02 | < .001*** | 0.63 | 0.56 | 0.71 |
| Education: High School | -0.24 | 0.05 | -5.27 | < .001*** | 0.78 | 0.71 | 0.86 |
| Education: Some College | -0.03 | 0.03 | -0.90 | .377 | 0.97 | 0.91 | 1.04 |
| Income: < $20,000 | 0.23 | 0.05 | 4.93 | < .001*** | 1.26 | 1.14 | 1.39 |
| Income: $20,000 - $49,999 | 0.16 | 0.03 | 4.65 | < .001*** | 1.17 | 1.09 | 1.26 |
| Income: $50,000 - $74,999 | 0.07 | 0.04 | 1.78 | .086 | 1.07 | 0.99 | 1.16 |
| Arrested during past 12 months | 1.12 | 0.05 | 24.33 | < .001*** | 3.06 | 2.79 | 3.36 |

**Sensitivity analysis for PR of past-year AUD using 2015-2019 data**

| Variable | *b** | *SE* | *t* | *p* | exp B | exp CI_lower | exp CI_upper |
| --- | --- | --- | --- | --- | --- | --- | --- |
| (Intercept) | -1.72 | 0.05 | -33.16 | < .001*** | 0.18 | 0.16 | 0.20 |
| Insurance: Medicaid/CHIP | -0.01 | 0.04 | -0.14 | .889 | 0.99 | 0.91 | 1.08 |
| Insurance: Medicare | -0.34 | 0.17 | -2.07 | .048* | 0.71 | 0.50 | 1.00 |
| Insurance: No insurance | -0.01 | 0.05 | -0.28 | .779 | 0.99 | 0.89 | 1.09 |
| Insurance: Other | -0.03 | 0.07 | -0.46 | .647 | 0.97 | 0.84 | 1.12 |
| Age: 26-34 | -0.24 | 0.03 | -7.70 | < .001*** | 0.78 | 0.73 | 0.84 |
| Age: 35-49 | -0.58 | 0.03 | -16.76 | < .001*** | 0.56 | 0.52 | 0.60 |
| Race: Black | -0.27 | 0.05 | -5.83 | < .001*** | 0.77 | 0.70 | 0.84 |
| Race: Hispanic | -0.29 | 0.04 | -7.48 | < .001*** | 0.75 | 0.69 | 0.81 |
| Race: Other | -0.33 | 0.06 | -5.47 | < .001*** | 0.72 | 0.63 | 0.81 |
| Metro: Non-Metro | -0.34 | 0.04 | -7.72 | < .001*** | 0.71 | 0.65 | 0.78 |
| Metro: Small Metro | -0.13 | 0.03 | -4.01 | < .001*** | 0.88 | 0.82 | 0.94 |
| Year: 2016 | -0.02 | 0.05 | -0.40 | .690 | 0.98 | 0.89 | 1.08 |
| Year: 2017 | -0.05 | 0.04 | -1.25 | .224 | 0.95 | 0.88 | 1.03 |
| Year: 2018 | -0.04 | 0.04 | -1.05 | .302 | 0.96 | 0.88 | 1.04 |
| Year: 2019 | -0.02 | 0.04 | -0.35 | .726 | 0.98 | 0.90 | 1.08 |
| Not pregnant, Parenting | -0.56 | 0.03 | -19.29 | < .001*** | 0.57 | 0.54 | 0.61 |
| Pregnant | -0.74 | 0.09 | -7.94 | < .001*** | 0.48 | 0.39 | 0.58 |
| Education: <High School | -0.44 | 0.05 | -8.27 | < .001*** | 0.64 | 0.58 | 0.72 |
| Education: High School | -0.18 | 0.05 | -3.44 | .002** | 0.84 | 0.75 | 0.93 |
| Education: Some College | -0.03 | 0.04 | -0.77 | .450 | 0.97 | 0.90 | 1.05 |
| Income: $20,000 | 0.18 | 0.05 | 3.63 | .001** | 1.19 | 1.08 | 1.32 |
| Income: $20,000 - $49,999 | 0.09 | 0.04 | 2.47 | .020* | 1.10 | 1.02 | 1.19 |
| Income: $50,000 - $74,999 | 0.03 | 0.04 | 0.67 | .511 | 1.03 | 0.95 | 1.11 |
| Arrested during the past 12 months | 1.16 | 0.05 | 23.07 | < .001*** | 3.18 | 2.87 | 3.52 |

**Sensitivity analysis for PR of past-year AUD using 2020-2021 data**

| Variable | *b** | *SE* | *t* | *p* | exp B | exp CI_lower | exp CI_upper |
| --- | --- | --- | --- | --- | --- | --- | --- |
| (Intercept) | -1.64 | 0.07 | -23.58 | < .001*** | 0.19 | 0.17 | 0.22 |
| Insurance: Medicaid/CHIP | -0.18 | 0.09 | -2.04 | .051 | 0.84 | 0.70 | 1.00 |
| Insurance: Medicare | -0.36 | 0.27 | -1.33 | .195 | 0.70 | 0.40 | 1.21 |
| Insurance: No insurance | 0.08 | 0.10 | 0.77 | .449 | 1.08 | 0.88 | 1.32 |
| Insurance: Other | 0.09 | 0.15 | 0.57 | .571 | 1.09 | 0.80 | 1.48 |
| Age: 26-34 | -0.03 | 0.07 | -0.48 | .631 | 0.97 | 0.85 | 1.11 |
| Age: 35-49 | -0.25 | 0.06 | -4.31 | < .001*** | 0.78 | 0.69 | 0.88 |
| Race: Black | -0.28 | 0.09 | -3.26 | .003** | 0.75 | 0.63 | 0.90 |
| Race: Hispanic | -0.42 | 0.09 | -4.64 | < .001*** | 0.66 | 0.55 | 0.79 |
| Race: Other | -0.38 | 0.11 | -3.38 | .002** | 0.68 | 0.54 | 0.86 |
| Metro: Non-Metro | -0.32 | 0.09 | -3.67 | .001*** | 0.73 | 0.61 | 0.87 |
| Metro: Small Metro | -0.10 | 0.06 | -1.78 | .085 | 0.90 | 0.80 | 1.01 |
| Year: 2020 | -0.01 | 0.05 | -0.20 | .843 | 0.99 | 0.89 | 1.10 |
| Not pregnant, Parenting | -0.33 | 0.06 | -5.16 | < .001*** | 0.72 | 0.63 | 0.82 |
| Pregnant | -0.56 | 0.16 | -3.40 | .002** | 0.57 | 0.41 | 0.80 |
| Education: <High School | -0.49 | 0.14 | -3.50 | .002** | 0.62 | 0.46 | 0.82 |
| Education: High School | -0.33 | 0.10 | -3.37 | .002** | 0.72 | 0.58 | 0.88 |
| Education: Some College | -0.01 | 0.07 | -0.13 | .899 | 0.99 | 0.86 | 1.15 |
| Income: < $20,000 | 0.32 | 0.10 | 3.39 | .002** | 1.38 | 1.14 | 1.68 |
| Income: $20,000 - $49,999 | 0.28 | 0.06 | 4.82 | < .001*** | 1.32 | 1.17 | 1.49 |
| Income: $50,000 - $74,999 | 0.14 | 0.09 | 1.55 | .132 | 1.15 | 0.96 | 1.38 |
| Arrested during the past 12 months | 1.00 | 0.14 | 7.33 | < .001*** | 2.72 | 2.06 | 3.59 |

**PR OF AUD TREATMENT**

**Bivariate models**

**Parenting status**

| Variable | *b** | *SE* | *t* | *p* | exp B | exp CI_lower | exp CI_upper |
| --- | --- | --- | --- | --- | --- | --- | --- |
| (Intercept) | -3.17 | 0.08 | -40.52 | < .001*** | 0.04 | 0.04 | 0.05 |
| Not pregnant, Parenting | 0.17 | 0.15 | 1.15 | .257 | 1.19 | 0.88 | 1.61 |
| Pregnant | 0.22 | 0.37 | 0.58 | .562 | 1.24 | 0.59 | 2.61 |

**Age**

| Variable | *b** | *SE* | *t* | *p* | exp B | exp CI_lower | exp CI_upper |
| --- | --- | --- | --- | --- | --- | --- | --- |
| (Intercept) | -3.40 | 0.11 | -31.97 | < .001*** | 0.03 | 0.03 | 0.04 |
| 26-34 | 0.39 | 0.17 | 2.25 | .029* | 1.47 | 1.04 | 2.09 |
| 35-49 | 0.47 | 0.15 | 3.17 | .003** | 1.59 | 1.19 | 2.14 |

**Race**

| Variable | *b** | *SE* | *t* | *p* | exp B | exp CI_lower | exp CI_upper |
| --- | --- | --- | --- | --- | --- | --- | --- |
| (Intercept) | -3.04 | 0.08 | -38.35 | < .001*** | 0.05 | 0.04 | 0.06 |
| Black | -0.14 | 0.20 | -0.69 | .493 | 0.87 | 0.58 | 1.31 |
| Hispanic | -0.13 | 0.18 | -0.75 | .457 | 0.88 | 0.61 | 1.25 |
| Other | -0.43 | 0.18 | -2.44 | .018* | 0.65 | 0.46 | 0.93 |

**Education**

| Variable | *b** | *SE* | *t* | *p* | exp B | exp CI_lower | exp CI_upper |
| --- | --- | --- | --- | --- | --- | --- | --- |
| (Intercept) | -3.59 | 0.14 | -24.92 | < .001*** | 0.03 | 0.02 | 0.04 |
| <High School | 1.06 | 0.20 | 5.25 | < .001*** | 2.88 | 1.92 | 4.32 |
| High School | 0.67 | 0.20 | 3.33 | .002** | 1.95 | 1.30 | 2.92 |
| Some College | 0.60 | 0.17 | 3.58 | .001*** | 1.81 | 1.30 | 2.54 |

**Income**

| Variable | *b** | *SE* | *t* | *p* | exp B | exp CI_lower | exp CI_upper |
| --- | --- | --- | --- | --- | --- | --- | --- |
| (Intercept) | -3.48 | 0.10 | -35.96 | < .001*** | 0.03 | 0.03 | 0.04 |
| < $20,000 | 0.83 | 0.16 | 5.29 | < .001*** | 2.29 | 1.67 | 3.14 |
| $20,000 - $49,999 | 0.37 | 0.17 | 2.24 | .030* | 1.45 | 1.04 | 2.03 |
| $50,000 - $74,999 | 0.28 | 0.16 | 1.76 | .086 | 1.33 | 0.96 | 1.83 |

**Insurance**

| Variable | *b** | *SE* | *t* | *p* | exp B | exp CI_lower | exp CI_upper |
| --- | --- | --- | --- | --- | --- | --- | --- |
| (Intercept) | -3.46 | 0.08 | -41.86 | < .001*** | 0.03 | 0.03 | 0.04 |
| Insurance: Medicaid/CHIP | 0.96 | 0.14 | 6.85 | < .001*** | 2.62 | 1.97 | 3.47 |
| Insurance: Medicare | 0.46 | 0.44 | 1.06 | .294 | 1.59 | 0.66 | 3.83 |
| Insurance: No insurance | 0.68 | 0.20 | 3.32 | .002** | 1.97 | 1.31 | 2.96 |
| Insurance: Other | 0.32 | 0.22 | 1.44 | .158 | 1.37 | 0.88 | 2.14 |

**History of arrests**

| Variable | *b** | *SE* | *t* | *p* | exp B | exp CI_lower | exp CI_upper |
| --- | --- | --- | --- | --- | --- | --- | --- |
| (Intercept) | -3.36 | 0.07 | -48.00 | < .001*** | 0.03 | 0.03 | 0.04 |
| Arrested during the past 12 months | 1.99 | 0.14 | 14.64 | < .001*** | 7.31 | 5.56 | 9.60 |

**Metro**

| Variable | *b** | *SE* | *t* | *p* | exp B | exp CI_lower | exp CI_upper |
| --- | --- | --- | --- | --- | --- | --- | --- |
| (Intercept) | -3.24 | 0.07 | -44.62 | < .001*** | 0.04 | 0.03 | 0.05 |
| Metro: Non-Metro | 0.36 | 0.21 | 1.73 | .090 | 1.43 | 0.94 | 2.18 |
| Metro: Small Metro | 0.29 | 0.12 | 2.32 | .025* | 1.33 | 1.04 | 1.70 |

**Year**

| Variable | *b** | *SE* | *t* | *p* | exp B | exp CI_lower | exp CI_upper |
| --- | --- | --- | --- | --- | --- | --- | --- |
| (Intercept) | -3.29 | 0.20 | -16.14 | < .001*** | 0.04 | 0.02 | 0.06 |
| 2015-2016 | 0.25 | 0.24 | 1.06 | .294 | 1.29 | 0.80 | 2.09 |
| 2017-2018 | 0.39 | 0.24 | 1.65 | .105 | 1.48 | 0.92 | 2.39 |
| 2019-2020 | 0.04 | 0.26 | 0.14 | .887 | 1.04 | 0.61 | 1.76 |

**PR of AUD treatment adjusted for non-SES variables**

| Variable | *b** | *SE* | *t* | *p* | exp B | exp CI_lower | exp CI_upper |
| --- | --- | --- | --- | --- | --- | --- | --- |
| (Intercept) | -3.84 | 0.21 | -18.11 | < .001*** | 0.02 | 0.01 | 0.03 |
| Age: 26-34 | 0.41 | 0.16 | 2.54 | .016* | 1.51 | 1.09 | 2.11 |
| Age: 35-49 | 0.53 | 0.15 | 3.53 | .001** | 1.70 | 1.25 | 2.31 |
| Race: Black | -0.21 | 0.21 | -1.00 | .322 | 0.81 | 0.53 | 1.24 |
| Race: Hispanic | -0.12 | 0.18 | -0.65 | .520 | 0.89 | 0.62 | 1.28 |
| Race: Other | -0.43 | 0.18 | -2.45 | .019* | 0.65 | 0.46 | 0.93 |
| Metro: Non-Metro | 0.21 | 0.22 | 0.95 | .349 | 1.23 | 0.79 | 1.93 |
| Metro: Small Metro | 0.24 | 0.11 | 2.13 | .040* | 1.27 | 1.01 | 1.59 |
| Not pregnant, Parenting | -0.01 | 0.16 | -0.09 | .925 | 0.99 | 0.72 | 1.35 |
| Pregnant | 0.06 | 0.34 | 0.18 | .854 | 1.06 | 0.54 | 2.12 |
| Year: 2015-2016 | 0.18 | 0.22 | 0.84 | .409 | 1.20 | 0.77 | 1.86 |
| Year: 2017-2018 | 0.31 | 0.23 | 1.37 | .180 | 1.36 | 0.86 | 2.15 |
| Year: 2019-2020 | -0.01 | 0.24 | -0.06 | .952 | 0.99 | 0.61 | 1.59 |
| Arrested during the past 12 months | 1.97 | 0.14 | 14.13 | < .001*** | 7.16 | 5.40 | 9.49 |

**PR of AUD treatment fully adjusted model**

| Variable | *b** | *SE* | *t* | *p* | exp B | exp CI_lower | exp CI_upper |
| --- | --- | --- | --- | --- | --- | --- | --- |
| (Intercept) | -4.28 | 0.21 | -20.73 | < .001*** | 0.01 | 0.01 | 0.02 |
| Not pregnant, Parenting | -0.11 | 0.14 | -0.75 | .462 | 0.90 | 0.67 | 1.21 |
| Pregnant | -0.07 | 0.34 | -0.20 | .840 | 0.93 | 0.47 | 1.86 |
| Education: <High School | 0.43 | 0.26 | 1.67 | .106 | 1.54 | 0.91 | 2.60 |
| Education: High School | 0.36 | 0.22 | 1.64 | .112 | 1.43 | 0.91 | 2.25 |
| Education: Some College | 0.45 | 0.19 | 2.35 | .026* | 1.56 | 1.06 | 2.30 |
| Income: < $20,000 | 0.28 | 0.20 | 1.39 | .177 | 1.33 | 0.87 | 2.02 |
| Income: $20,000 - $49,999 | 0.00 | 0.21 | 0.01 | .989 | 1.00 | 0.65 | 1.55 |
| Income: $50,000 - $74,999 | 0.13 | 0.17 | 0.78 | .443 | 1.14 | 0.81 | 1.62 |
| Insurance: Medicaid/CHIP | 0.52 | 0.15 | 3.45 | .002** | 1.67 | 1.23 | 2.28 |
| Insurance: Medicare | -0.26 | 0.40 | -0.65 | .519 | 0.77 | 0.34 | 1.74 |
| Insurance: No insurance | 0.32 | 0.20 | 1.57 | .129 | 1.38 | 0.91 | 2.09 |
| Insurance: Other | 0.29 | 0.23 | 1.26 | .218 | 1.34 | 0.83 | 2.14 |
| Age: 26-34 | 0.46 | 0.17 | 2.79 | .009** | 1.59 | 1.13 | 2.23 |
| Age: 35-49 | 0.65 | 0.16 | 4.07 | < .001*** | 1.91 | 1.38 | 2.64 |
| Race: Black | -0.42 | 0.21 | -1.98 | .058 | 0.66 | 0.42 | 1.02 |
| Race: Hispanic | -0.28 | 0.20 | -1.38 | .179 | 0.76 | 0.50 | 1.14 |
| Race: Other | -0.50 | 0.17 | -2.93 | .007** | 0.60 | 0.42 | 0.86 |
| Metro: Non-Metro | 0.03 | 0.22 | 0.14 | .891 | 1.03 | 0.66 | 1.61 |
| Metro: Small Metro | 0.16 | 0.13 | 1.25 | .223 | 1.17 | 0.90 | 1.52 |
| Arrested during the past 12 months | 1.68 | 0.15 | 11.01 | < .001*** | 5.37 | 3.92 | 7.34 |
| Year: 2015-2016 | 0.15 | 0.21 | 0.71 | .481 | 1.16 | 0.75 | 1.81 |
| Year: 2017-2018 | 0.31 | 0.22 | 1.39 | .176 | 1.36 | 0.86 | 2.16 |
| Year: 2019-2020 | -0.02 | 0.23 | -0.08 | .934 | 0.98 | 0.61 | 1.59 |

**Sensitivity analysis for PR of AUD treatment using 2015-2019 data**

| Variable | *b** | *SE* | *t* | *p* | exp B | exp CI_lower | exp CI_upper |
| --- | --- | --- | --- | --- | --- | --- | --- |
| (Intercept) | -4.13 | 0.25 | -16.64 | < .001*** | 0.02 | 0.01 | 0.03 |
| Not pregnant, Parenting | -0.00 | 0.15 | -0.00 | .997 | 1.00 | 0.74 | 1.35 |
| Pregnant | -0.26 | 0.39 | -0.66 | .513 | 0.77 | 0.35 | 1.71 |
| Education: <High School | 0.46 | 0.21 | 2.18 | .039* | 1.58 | 1.03 | 2.42 |
| Education: High School | 0.27 | 0.19 | 1.42 | .167 | 1.31 | 0.89 | 1.95 |
| Education: Some College | 0.48 | 0.17 | 2.85 | .009** | 1.61 | 1.14 | 2.28 |
| Income: < $20,000 | -0.02 | 0.20 | -0.10 | .923 | 0.98 | 0.65 | 1.47 |
| Income: $20,000 - $49,999 | -0.20 | 0.20 | -0.96 | .344 | 0.82 | 0.54 | 1.25 |
| Income: $50,000 - $74,999 | 0.12 | 0.18 | 0.65 | .520 | 1.13 | 0.77 | 1.64 |
| Insurance: Medicaid/CHIP | 0.51 | 0.17 | 2.97 | .006** | 1.66 | 1.17 | 2.37 |
| Insurance: Medicare | -0.33 | 0.56 | -0.59 | .558 | 0.72 | 0.22 | 2.28 |
| Insurance: No insurance | 0.28 | 0.20 | 1.40 | .174 | 1.33 | 0.87 | 2.02 |
| Insurance: Other | 0.34 | 0.25 | 1.40 | .172 | 1.41 | 0.85 | 2.34 |
| Age: 26-34 | 0.46 | 0.15 | 3.08 | .005** | 1.58 | 1.16 | 2.14 |
| Age: 35-49 | 0.65 | 0.14 | 4.58 | < .001*** | 1.92 | 1.43 | 2.58 |
| Race: Black | -0.40 | 0.22 | -1.87 | .073 | 0.67 | 0.43 | 1.04 |
| Race: Hispanic | -0.25 | 0.23 | -1.08 | .290 | 0.78 | 0.48 | 1.26 |
| Race: Other | -0.57 | 0.25 | -2.31 | .029* | 0.57 | 0.34 | 0.94 |
| Metro: Non-Metro | -0.20 | 0.20 | -0.98 | .338 | 0.82 | 0.54 | 1.25 |
| Metro: Small Metro | 0.05 | 0.14 | 0.33 | .743 | 1.05 | 0.79 | 1.39 |
| Arrested during the past 12 months | 1.64 | 0.17 | 9.80 | < .001*** | 5.16 | 3.66 | 7.28 |
| Year: 2016 | 0.36 | 0.26 | 1.39 | .177 | 1.43 | 0.84 | 2.45 |
| Year: 2017 | 0.30 | 0.25 | 1.22 | .233 | 1.36 | 0.81 | 2.26 |
| Year: 2018 | 0.38 | 0.21 | 1.82 | .081 | 1.47 | 0.95 | 2.27 |
| Year: 2019 | 0.31 | 0.20 | 1.60 | .122 | 1.37 | 0.91 | 2.05 |

**Sensitivity analysis for PR of AUD treatment using 2020-2021 data**

| Variable | *b** | *SE* | *t* | *p* | exp B | exp CI_lower | exp CI_upper |
| --- | --- | --- | --- | --- | --- | --- | --- |
| (Intercept) | -5.06 | 0.51 | -9.97 | < .001*** | 0.01 | 0.00 | 0.02 |
| Not pregnant, Parenting | -0.33 | 0.32 | -1.02 | .317 | 0.72 | 0.37 | 1.39 |
| Pregnant | 0.47 | 0.62 | 0.76 | .451 | 1.60 | 0.45 | 5.69 |
| Education: <High School | 0.08 | 0.77 | 0.11 | .913 | 1.09 | 0.23 | 5.23 |
| Education: High School | 0.31 | 0.56 | 0.56 | .581 | 1.37 | 0.43 | 4.35 |
| Education: Some College | 0.17 | 0.55 | 0.31 | .762 | 1.18 | 0.39 | 3.63 |
| Income: < $20,000 | 1.38 | 0.46 | 2.97 | .006** | 3.98 | 1.54 | 10.27 |
| Income: $20,000 - $49,999 | 0.78 | 0.58 | 1.34 | .191 | 2.19 | 0.66 | 7.22 |
| Income: $50,000 - $74,999 | 0.21 | 0.58 | 0.37 | .716 | 1.24 | 0.38 | 4.03 |
| Insurance: Medicaid/CHIP | 0.46 | 0.30 | 1.53 | .138 | 1.59 | 0.85 | 2.97 |
| Insurance: Medicare | -0.18 | 0.63 | -0.28 | .779 | 0.84 | 0.23 | 3.02 |
| Insurance: No insurance | 0.47 | 0.38 | 1.21 | .234 | 1.59 | 0.73 | 3.50 |
| Insurance: Other | 0.36 | 0.64 | 0.56 | .581 | 1.43 | 0.39 | 5.26 |
| Age: 26-34 | 0.43 | 0.43 | 1.01 | .322 | 1.54 | 0.64 | 3.73 |
| Age: 35-49 | 0.66 | 0.44 | 1.51 | .143 | 1.94 | 0.79 | 4.77 |
| Race: Black | -0.32 | 0.47 | -0.68 | .499 | 0.73 | 0.28 | 1.88 |
| Race: Hispanic | -0.22 | 0.42 | -0.53 | .602 | 0.80 | 0.34 | 1.90 |
| Race: Other | -0.33 | 0.42 | -0.78 | .443 | 0.72 | 0.30 | 1.71 |
| Metro: Non-Metro | 0.66 | 0.44 | 1.50 | .143 | 1.94 | 0.79 | 4.75 |
| Metro: Small Metro | 0.61 | 0.27 | 2.29 | .029* | 1.85 | 1.07 | 3.19 |
| Arrested during the past 12 months | 1.66 | 0.35 | 4.75 | < .001*** | 5.27 | 2.57 | 10.77 |
| Year: 2020 | -0.35 | 0.33 | -1.05 | .302 | 0.71 | 0.36 | 1.39 |

**Barrier to AUD treatment distribution across by parenting status**


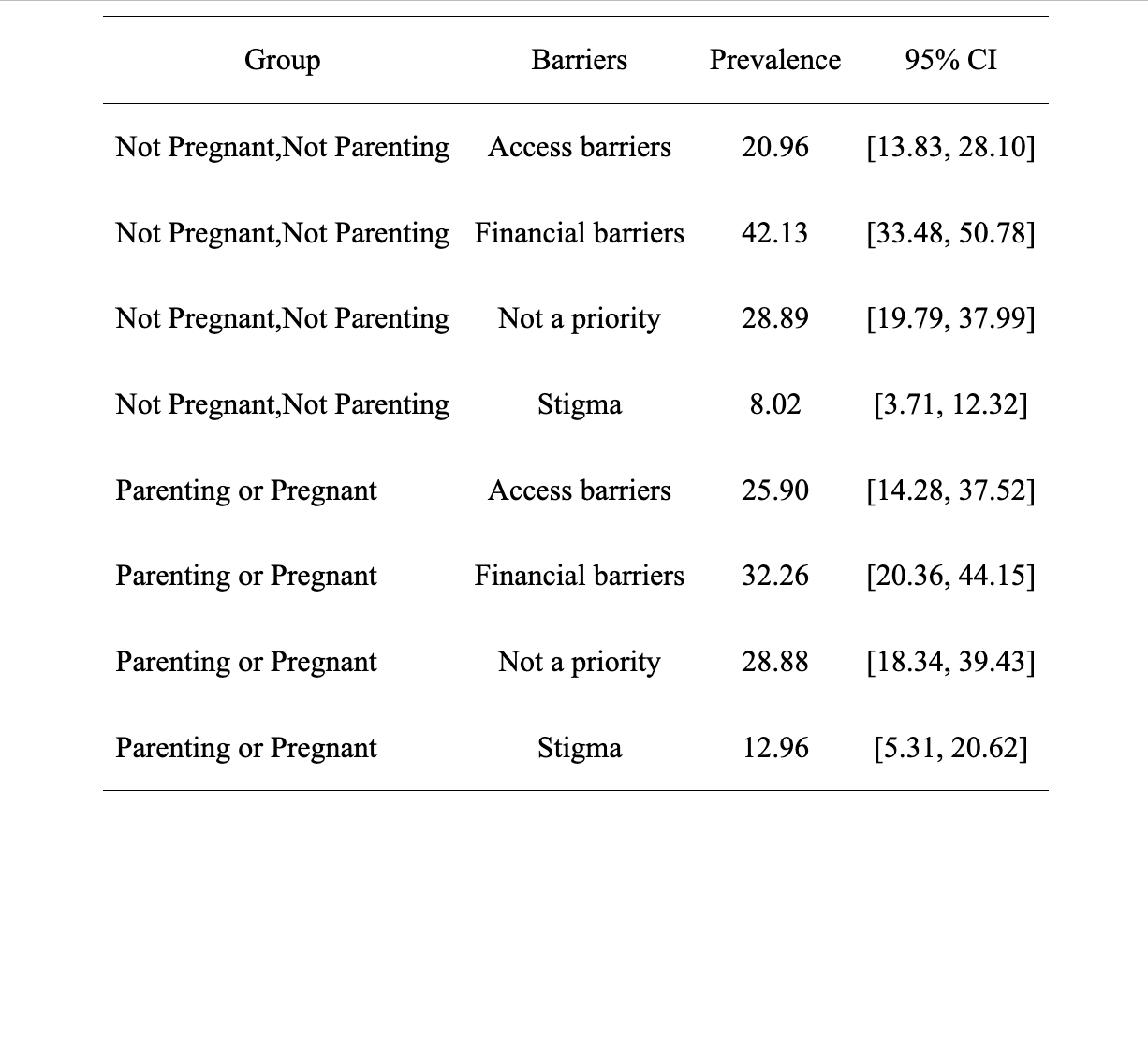

Supplement: S5 Table — (DOCX) [file pone.0301810.s005.docx]
